# Supplementary material for: Developing and validating an explainable digital mortality prediction tool for extremely preterm infants
Source: PLOS Digit Health. 2025 Dec 10;4(12):e0000955. doi: 10.1371/journal.pdig.0000955 (PMC12694798; doi:10.1371/journal.pdig.0000955)
Supplement: S1 Table — IQR denotes interquartile range. (DOCX) [file pdig.0000955.s001.docx]

# S1 Table

Table demonstrating the characteristics of infants with complete data included in developing and validating the prediction model, and excluded infants with missing data for birth weight z score, multiple pregnancy, exposure to antenatal corticosteroids or born in a centre with neonatal intensive care unit (NICU).

IQR denotes interquartile range.

| **Clinical characteristics** | **Complete data**  **(N=24,260)** | **Missing data**  **(N = 1,642)** |
| --- | --- | --- |
| **Gestation at birth** (weeks), median (IQR) | 26^+1^ (24^+6^ to 27^+1^) | 26^+0^ (24^+4^ to 27^+0^) |
| **Birth weight Z score^1^**, median (IQR)  Missing, n (%) | -0.39 (-0.91 to 0.08)  0 (0) | -0.29 (-0.77 to 0.13)  1 (0.06) |
| **Sex**, n (%) |  |  |
| Male  Missing | 13,220 (54)  0 (0) | 862 (53)  1 (0.06) |
| **Multiple pregnancy**, n (%)  Missing | 5,925 (24)  0 (0) | 338 (21)  3 (0.2) |
| **Antenatal corticosteroids**, n (%)  Complete course  Incomplete course  No course  Missing | 17,227 (71)  5,146 (21)  1,887 (8)  0 (0) | 3 (0.2)  1 (0.06)  0 (0)  1,638 (100) |
| **Congenital anomaly**, n (%) | 654 (3) | 46 (3) |
| **Chorioamnionitis**, n (%) | 1,929 (8) | 51 (3) |
| **Prolonged rupture of membranes**, n (%) | 3,280 (14) | 96 (6) |
| **Born in a centre with NICU**, n (%)  Missing | 16,970 (70)  0 (0) | 1,049 (64)  3 (0.2) |
| **Maternal ethnicity**, n (%)  White  South Asian  Black  Others/Mix  Missing | 14,401 (59)  2,807 (12)  2,554 (11)  886 (4)  3,612 (15) | 902 (55)  191 (12)  140 (9)  77 (5)  332 (20) |
| **Neonatal network at birth**, n (%)  Network 1  Network 2  Network 3  Network 4  Network 5  Network 6  Network 7  Network 8  Network 9  Network 10  Network 11  Network 12  Network 13  Missing | 1,269 (5)  1,889 (8)  2,119 (9)  1,367 (6)  3,301 (14)  1,252 (5)  1,889 (8)  1,769 (7)  1,463 (6)  1,988 (8)  760 (3)  2,598 (11)  2,177 (9)  419 (2) | 186 (11)  99 (6)  94 (6)  56 (3)  251 (15)  74 (5)  76 (5)  89 (5)  94 (6)  99 (6)  45 (3)  199 (12)  110 (7)  170 (10) |
| **Infant death before discharge**, n (%) | 5,039 (21) | 511 (31) |
