# Supplementary material for: Developing and validating an explainable digital mortality prediction tool for extremely preterm infants
Source: PLOS Digit Health. 2025 Dec 10;4(12):e0000955. doi: 10.1371/journal.pdig.0000955 (PMC12694798; doi:10.1371/journal.pdig.0000955)
Supplement: S1 File — (DOCX) [file pdig.0000955.s002.docx]

# S1 File

File detailing the model development process for each of the nine machine learning approaches.

**Overall cohort**

25,902 infants

**Training cohort**

**(2010–2015)**

14,535 infants

**Validation cohort**

**(2016–2017)**

4,871 infants

**Development cohort (2010–2017)**

19,406 infants

**Test cohort**

**(2018–2020)**

6,496 infants

For the logistic regression and AutoPrognosis 2.0 approaches, infants born from 2010 to 2017 were used to train the models. For the subsequent seven machine learning approaches, the model was trained on the cohort of infants born from 2010 to 2015 (‘training’ cohort) using different hyperparameters. Infants born from 2016 to 2017 (‘validation’ cohort) were then used to identify the optimal hyperparameters that produced the best discrimination performance for each of the seven machine learning approaches. The dataset was z-score normalised based on the training data values to ensure stability and speed up the convergence of these seven machine learning approaches.

## Logistic Regression

Based on the methodology proposed by Riley et al(51) and the performance of a previously published model(2), a minimum sample size of 1,520 infants is needed to develop a logistic regression model with 15 predictors. Stepwise backward regression^(52)^, with variables included if p-value <0.05, was performed using the glm and MASS packages(53) in R. Chorioamnionitis and congenital anomaly variables were excluded from the final model (**Supplementary Table 1**).

Restricted cubic splines(54) were used to flexibly model the continuous variables of gestational age at birth due to its non-linearity with the logit function of mortality risk. Restricted cubic spline was chosen as it is constrained to be linear at the two tails (before the first knot and after the last knot), avoiding a poor fit for extreme observations(55). Four knots (23^+5^, 25^+3^, 26^+5^, and 27^+5^ weeks) were chosen as they produced the optimal Akaike Information Criterion when choosing between three, four and five knots.

Scatterplot of the observed logit transformation of mortality in deciles (red circles) with gestational age at birth assuming linear relationship (dotted blue line) and after restricted cubic spline transformation (solid black line) of gestational age at birth at the respective four knots of 23^+5^, 25^+3^, 26^+5^, and 27^+5^ weeks (dotted vertical grey line).

Within the final model (**Supplementary Table 1**), there was no evidence of multicollinearity, with all predictors having a variance inflation factor of below 10. There were also no significant outliers, with only 4.3% and 0.04% of the standardised residuals outside ±1.96 and ±2.58, respectively.

| **Predictors** | **Variance inflation factor** |
| --- | --- |
| Gestation at birth | 1.04 |
| Birth weight z score | 1.05 |
| Sex | 1.00 |
| Antenatal corticosteroids | 1.17 |
| Multiple pregnancy | 1.01 |
| Prolonged rupture of membranes | 1.05 |
| Born in a centre with a NICU | 1.12 |

## AutoPrognosis 2.0

AutoPrognosis 2.0(56) Python module was used to train the AutoPrognosis 2.0 model in R. AutoPrognosis 2.0(56) is an automated machine learning framework that automatically configures and optimises machine learning pipelines using state-of-the-art algorithms. All available classifiers in AutoPrognosis 2.0 were used, which included AdaBoost, Bagging, Bernoulli Naïve Bayes, CatBoost, Decision Tree, Extra Tree Classifier, Gaussian Naïve Bayes, Gaussian Process, Gradient Boosting, Histogram-based Gradient Boosting, K-Nearest Neighbour, Linear Discriminant Analysis, Light Gradient Boosting Machine, Linear Support Vector Machine, Logistic Regression, Multinomial Naïve Bayes, Neural Network, Perceptron, Quadratic Discriminant Analysis, Random Forest, Ridge Classifier, TabNet and Extreme Gradient Boosting. The best model from AutoPrognosis 2.0 was an aggregate ensemble model of Gaussian Process, Gaussian Naïve Bayes and Light Gradient Boosting Machine.

## Adaptive Neuro-Fuzzy Inference System (ANFIS)

If-Then rules were first developed a-priori based on clinical expertise.

| **Gestation** | **Risk of death** |
| --- | --- |
| **< 24^+0^** | Very high |
| **24^+0^ to 25^+6^** | High |
| **26^+0^ to 27^+6^** | Moderate |

| **Birth weight z score** | **Risk of death** |
| --- | --- |
| **< -1.28** | High |
| **-1.28 to -0.66** | Moderate |
| **-0.67 to 0.66** | Low |
| **0.67 to 1.28** | Moderate |
| **>1.28** | High |

1. If the exposure to antenatal corticosteroids is ‘complete course’ and the gestation is 24^+0^ to 25^+6^, the risk of death is ‘moderate’.
2. If the exposure to antenatal corticosteroids is ‘complete course’ and the gestation is <24^+0^, the risk of death is ‘high.
3. If the sex is ‘female’, the gestation is 24^+0^ to 25^+6^ weeks, and the birth weight z score is ≤-0.66, the risk of death is ‘moderate’.
4. If the congenital anomaly is ‘yes’ and the birth weight z score is -0.67 to 0.66, the risk of death is ‘high’.
5. If the prolonged rupture of membranes is ‘yes’, the gestation is ≤27^+6^ weeks and the birth weight z score is ≤-0.66, the risk of death is ‘very high’.
6. If the prolonged rupture of membranes is ‘yes’, the gestation is 24^+0^ to 27^+6^ weeks and the birth weight z score is -0.67 to 0.66, the risk of death is ‘high’.
7. If born in a centre with a neonatal intensive care unit is ‘yes’ and the gestation is 24^+0^ to 25^+6^ weeks, the risk of death is ‘moderate’.
8. If born in a centre with a neonatal intensive care unit is ‘yes’ and the birth weight z score is <-1.28, the risk of death is ‘moderate’.
9. If multiple pregnancy is ‘yes’ and the gestation is ≤27^+6^ weeks, the risk of death is ‘high’.
10. If multiple pregnancy is ‘yes’ and the birth weight z score is ≤-0.66, the risk of death is ‘high’.
11. If chorioamnionitis is ‘yes’, the gestation is ≤ 27^+6^ weeks and the birth weight z score is ≤-0.66, the risk of death is ‘very high’.
12. If chorioamnionitis is ‘yes’, the gestation is 24^+0^ to 27^+6^ weeks and the birth weight z score is -0.67 to 0.66, the risk of death is ‘high’.

FuzzyR package(57) was used in R to develop the model. Various combinations of predictors (sex, exposure to antenatal corticosteroids and multiple pregnancy) were explored to group the dataset for the ANFIS approach. In the final ANFIS model, sex and multiple pregnancy predictors were used to group the dataset. 200 training epochs with 4 randomly selected rules, a step size of 0.3 and T1 membership function type were used.

## Extreme Gradient Boosting (XGBoost)

XGBoost XGBClassifier module(58) in Python was used to develop the model. Hyperparameters for the number of boosting rounds, maximum tree depth for base learners, boosting learning rate, subsample ratio of columns when constructing each tree, subsample ratio of the training instance, and global bias were determined from a range of 300 to 800, 3 to 8, 0.001 to 0.01, 0.4 to 7, 0.1 to 1 and 0.4 to 9 respectively using the “GridSearchCV” function. The best model had 600 boosting rounds, a maximal tree depth for base learners of 3, 0.009 boosting learning rate, a subsample ratio of columns when constructing each tree of 1, a subsample ratio of the training instances of 0.1 and 0.9 global bias.

## Feedforward neural network

Keras Application Programming Interface(59) in Python with 50 training epochs was used to develop the model. Hyperparameters for the number of layers, the number of nodes within each layer, and the learning rate were determined from a range of 1 to 5, 3 to 45, and 0.0001 to 0.01, respectively, using the “KerasTuner RandomSearch” function. The optimisation algorithm was chosen between the rmsprop and adam optimiser. The activation function was chosen between Sigmoid, Tanh or Rectified Linear Unit (ReLU). The best model has five dense layers with 9, 24, 18, 9 and 1 nodes respectively using the adam optimiser, Sigmoid activation, and a learning rate of 0.001.

## K-Nearest Neighbour

Caret package(60) in R was used to develop the model. The hyperparameter for the k-value was determined from a range of 1 to 40 using the “trainControl” function. The best model has a k value of 40.

## Long Short-Term Memory

Keras Application Programming Interface(59) in R with 200 training epochs was used to develop the model. The data within the ‘training’ cohort was first ordered by birth year. The number of layers, the number of nodes within each layer (3, 5, 6 or 9), the batch size (8, 16, 32, 64, 128 or 512), the choice of the optimisation algorithm (rmsprop or adam), and the activation function (Sigmoid or Tanh or ReLU) were determined based on the performance in the ‘validation’ cohort. The final model has four layers consisting of a LSTM layer with 9 nodes and Tanh activation, followed by two dense layers with 5 nodes and ReLU activation, as well as a last dense layer with 1 node and Sigmoid activation. The model used the adam optimiser and a batch size of 512.

## Random Forest

Scikit-learn Random Forest Classifier module(61) in Python was used to develop the model. Hyperparameters for the number of trees and the maximal depth of the tree were determined from a range of 100 to 1000 and 3 to 10, respectively, using the “GridSearchCV”. The best model had a number of trees of 100 and a maximal depth of the tree of 7.

## Radial Kernel Support Vector Machine (SVM)

Caret package(60) in R was used to develop the model. Hyperparameters for the sigma and c values were determined from a range of 0 to 0.9 and 0 to 5, respectively, using the “trainControl” function. The best model has a sigma and c values of 0.05 and 5, respectively.

## Summary Table

Table summarising the machine learning approaches used.

| **Machine learning approach** | **Package used** | **Hyperparameter/Final algorithm used** |
| --- | --- | --- |
| **Logistic regression** | glm and MASS package(53) in R | - Stepwise backward regression^(52)^, with variables included if p-value <0.05. - Chorioamnionitis and congenital anomaly variables were excluded from the final model (**Supplementary Table 3**). - Restricted cubic splines^(54)^ to model the continuous variables of gestational age at birth with four knots (23^+5^, 25^+3^, 26^+5^, and 27^+5^ weeks). |
| **AutoPrognosis 2.0** | Autoporgnosis 2.0(56) in R | - Aggregate ensemble model of Gaussian Process, Gaussian Naïve Bayes and Light Gradient Boosting Machine. |
| **Adaptive Neuro-Fuzzy Inference System (ANFIS)** | FuzzyR package(57) in R | - A-priori determined If-Then Rules (refer to above). - Sex and multiple pregnancy predictors were used to group the dataset. - 200 training epochs, 0.3 step size, T1 membership function type. |
| **Extreme Gradient Boosting (XGBoost)** | XGBoost XGBClassifier module(58) in Python | - Hyperparameters were determined using “GridSearchCV” function. - 600 boosting rounds, maximal tree depth for base learners of 3, 0.009 boosting learning rate, subsample ratio of columns when constructing each tree of 1, subsample ratio of the training instances of 0.1 and 0.9 global bias. |
| **Feedforward neural network** | Keras Application Programming Interface(59) in Python | - Hyperparameters were determined using “KerasTuner RandomSearch” function. - Five dense layers of 9, 24, 18, 9 and 1 nodes respectively and Sigmoid activation. - Adam optimiser, 50 training epochs, 0.001 learning rate. |
| **K-Nearest Neighbour** | Caret package(60) in R | - Hyperparameters were determined using “trainControl” function. - k value of 40. |
| **Long Short-Term Memory (LSTM)** | Keras Application Programming Interface(59) in R | - Data within the ‘training’ cohort was ordered by birth year. - Four layers of LSTM layer of 9 nodes and Tanh activation; two dense layers of 5 nodes and Rectified Linear Unit (ReLU) activation; and one dense layer of 1 node and Sigmoid activation. - Adam optimiser, 200 training epochs, 512 batch size. |
| **Random Forest** | Scikit-learn Random Forest Classifier module(61) in Python | - Hyperparameters were determined using “GridSearchCV” function. - 100 trees and maximal tree depth of 7. |
| **Radial Kernel Support Vector Machine (SVM)** | Caret package(60) in R | - Hyperparameters were determined using “trainControl” function. - Sigma value of 0.05 and c value of 5. |

## References

1. Riley RD, Ensor J, Snell KIE, et al. Calculating the sample size required for developing a clinical prediction model. *BMJ* 2020; **368**: m441.

2. Santhakumaran S, Statnikov Y, Gray D, et al. Survival of very preterm infants admitted to neonatal care in England 2008-2014: time trends and regional variation. *Arch Dis Child Fetal Neonatal Ed* 2018; **103**(3): F208-F15.

3. Royston PS, W. Multivariable model-building. A pragmatic approach to regression analysis based on fractional polynomials for modelling continuous variables. Chichester: John Wiley & Sons Ltd; 2008.

4. Venables WN, Ripley BD. Modern Applied Statistics with S. Fourth ed. New York: Springer; 2002.

5. Harrell FE. Regression Modeling Strategies: With Applications to Linear Models, Logistic and Ordinal Regression, and Survival Analysis, 2nd Edition. *Regression Modeling Strategies: with Applications to Linear Models, Logistic and Ordinal Regression, and Survival Analysis 2*015.

6. Durrleman S, Simon R. Flexible regression models with cubic splines. *Statistics in Medicine* 1989; **8**(5): 551-61.

7. Imrie F, Cebere B, McKinney EF, van der Schaar M. AutoPrognosis 2.0: Democratizing diagnostic and prognostic modeling in healthcare with automated machine learning. *PLOS Digital Health* 2023; **2**(6): e0000276.

8. Chen C, John R, Twycross J, Garibaldi JM. An extended ANFIS architecture and its learning properties for type-1 and interval type-2 models. 2016 IEEE International Conference on Fuzzy Systems (FUZZ-IEEE); 2016 24-29 July 2016; 2016. p. 602-9.

9. Chen T, Guestrin C. XGBoost: A Scalable Tree Boosting System. 22nd ACM SIGKDD International Conference on Knowledge Discovery and Data Mining; 2016; San Francisco, California, USA: ACM; 2016. p. 785–94.

10. JJ Allaire and François Chollet (2020). keras: R Interface to 'Keras'. R package version 2.3.0.0.  <https://CRAN.R-project.org/package=keras>

11. Kuhn M. Building Predictive Models in R Using the caret Package. *Journal of Statistical Software* 2008; **28**(5): 1-26.

12. Pedregosa FVG, Gramfort A, Michel V, Thirion B, Grisel O, Blondel M, Prettenhofer P, Weiss R, Dubourg V, Vanderplas J, Passos A, Cournapeau D, Brucher M, Perrot M, Duchesnay E. Scikit-learn: Machine Learning in Python. *Journal of Machine Learning Research* 2011; **12**: 2825-30.
