# Supplementary material for: Developing and validating an explainable digital mortality prediction tool for extremely preterm infants
Source: PLOS Digit Health. 2025 Dec 10;4(12):e0000955. doi: 10.1371/journal.pdig.0000955 (PMC12694798; doi:10.1371/journal.pdig.0000955)
Supplement: S2 Table — NICU denotes Neonatal Intensive Care Unit. PROM denotes Prolonged rupture of membranes. 1 Chorioamnionitis and congenital anomalies predictors were dropped from the logistic regression model after backward stepwise selection. 2 Sex and Multiple pregnancy predictors were used to group the dataset in the Adaptive Neuro-Fuzzy Inference System. (DOCX) [file pdig.0000955.s003.docx]

# S2 Table

Table demonstrating the importance of the predictors to the predictions in the testing dataset based on mean SHapley Additive exPlanations (SHAP) values. NICU denotes Neonatal Intensive Care Unit. PROM denotes Prolonged rupture of membranes.

^1^ Chorioamnionitis and congenital anomalies predictors were dropped from the logistic regression model after backward stepwise selection

^2^ Sex and Multiple pregnancy predictors were used to group the dataset in the Adaptive Neuro-Fuzzy Inference System.

| **(A) Logistic Regression^1^** | | **(B) Adaptive Neuro-Fuzzy Inference System^2^** | | **(C) AutoPrognosis 2.0** | |
| --- | --- | --- | --- | --- | --- |
| **Predictor** | **Mean SHAP value** | **Predictor** | **Mean SHAP value** | **Predictor** | **Mean SHAP value** |
| Gestational age at birth | 0.690 | Gestational age at birth | 0.119 | Gestational age at birth | 0.114 |
| Birth weight z-score | 0.226 | Birth weight z-score | 0.097 | Birth weight z-score | 0.024 |
| Antenatal corticosteroids | 0.114 | Chorioamnionitis | 0.053 | Antenatal corticosteroids | 0.023 |
| Sex | 0.114 | PROM | 0.032 | Multiple Pregnancy | 0.012 |
| Born in centre with NICU | 0.070 | Antenatal corticosteroids | 0.027 | Born in centre with NICU | 0.011 |
| Multiple Pregnancy | 0.065 | Born in centre with NICU | 0.018 | PROM | 0.009 |
| PROM | 0.042 | Congenital Anomalies | 0.013 | Sex | 0.008 |
| Chorioamnionitis | N/A | Sex | N/A | Chorioamnionitis | 0.001 |
| Congenital Anomalies | N/A | Multiple Pregnancy | N/A | Congenital Anomalies | 0.001 |

| **(D) Extreme Gradient Boosting** | | **(E) Feedforward Neural Network** | | **(F) K-Nearest Neighbour** | |
| --- | --- | --- | --- | --- | --- |
| **Predictor** | **Mean SHAP value** | **Predictor** | **Mean SHAP value** | **Predictor** | **Mean SHAP value** |
| Gestational age at birth | 0.120 | Gestational age at birth | 0.111 | Gestational age at birth | 0.102 |
| Birth weight z-score | 0.039 | Birth weight z-score | 0.035 | Birth weight z-score | 0.034 |
| Antenatal corticosteroids | 0.014 | Antenatal corticosteroids | 0.025 | Sex | 0.020 |
| Sex | 0.011 | Sex | 0.017 | Antenatal corticosteroids | 0.014 |
| Multiple Pregnancy | 0.009 | Born in centre with NICU | 0.010 | Multiple Pregnancy | 0.014 |
| Born in centre with NICU | 0.007 | Multiple Pregnancy | 0.010 | Born in centre with NICU | 0.008 |
| PROM | 0.005 | PROM | 0.009 | PROM | 0.007 |
| Congenital Anomalies | 0.002 | Chorioamnionitis | 0.003 | Chorioamnionitis | 0.006 |
| Chorioamnionitis | 0.000 | Congenital Anomalies | 0.002 | Congenital Anomalies | 0.006 |

| **(G) Long Short-Term Memory** | | **(H) Random Forest** | | **(I) Support Vector Machine** | |
| --- | --- | --- | --- | --- | --- |
| **Predictor** | **Mean SHAP value** | **Predictor** | **Mean SHAP value** | **Predictor** | **Mean SHAP value** |
| Gestational age at birth | 0.102 | Gestational age at birth | 0.104 | Gestational age at birth | 0.037 |
| Birth weight z-score | 0.040 | Birth weight z-score | 0.030 | Antenatal corticosteroids | 0.018 |
| Antenatal corticosteroids | 0.014 | Antenatal corticosteroids | 0.012 | Birth weight z-score | 0.012 |
| Born in centre with NICU | 0.008 | Sex | 0.010 | Multiple Pregnancy | 0.007 |
| Multiple Pregnancy | 0.007 | Multiple Pregnancy | 0.006 | Born in centre with NICU | 0.006 |
| Sex | 0.005 | Born in centre with NICU | 0.003 | Sex | 0.005 |
| PROM | 0.005 | PROM | 0.003 | PROM | 0.002 |
| Chorioamnionitis | 0.003 | Congenital Anomalies | 0.002 | Chorioamnionitis | 0.003 |
| Congenital Anomalies | 0.003 | Chorioamnionitis | 0.001 | Congenital Anomalies | 0.003 |
