# Supplementary material for: Developing and validating an explainable digital mortality prediction tool for extremely preterm infants
Source: PLOS Digit Health. 2025 Dec 10;4(12):e0000955. doi: 10.1371/journal.pdig.0000955 (PMC12694798; doi:10.1371/journal.pdig.0000955)
Supplement: S1 Fig — (DOCX) [file pdig.0000955.s004.docx]

# S1 Fig

Figure demonstrating the importance of the predictors to the predictions of the logistic regression model in the ‘test’ cohort based on mean SHapley Additive exPlanations (SHAP) values for the overall cohort and by gestational week groups.
