# Supplementary material for: Developing and validating an explainable digital mortality prediction tool for extremely preterm infants
Source: PLOS Digit Health. 2025 Dec 10;4(12):e0000955. doi: 10.1371/journal.pdig.0000955 (PMC12694798; doi:10.1371/journal.pdig.0000955)
Supplement: S3 Table — (DOCX) [file pdig.0000955.s005.docx]

# S3 Table

Table describing the distribution of the predicted risk obtained from the nine machine learning approaches used in the ‘test’ cohort (N = 5,879).

| **Predicted risk** | **Extreme Gradient Boosting** | **Feedforward Neural Network** | **Random Forest** | **Long Short-Term Memory** | **Adaptive Neuro-Fuzzy Inference System** | **AutoPrognosis 2.0** | **Logistic Regression** | **K-Nearest Neighbour** | **Support Vector Machine** |
| --- | --- | --- | --- | --- | --- | --- | --- | --- | --- |
| <0.2 | 3,585 (61%) | 3,694 (63%) | 3,704 (63%) | 3,777 (64%) | 3,539 (60%) | 3,680 (63%) | 3,702 (63%) | 3,418 (58%) | 4,978 (85%) |
| 0.2 - <0.4 | 1,427 (24%) | 1,474 (25%) | 1,397 (24%) | 1,516 (26%) | 1,435 (24%) | 1,500 (26%) | 1,419 (24%) | 1,730 (29%) | 685 (12%) |
| 0.4 - <0.6 | 680 (12%) | 598 (10%) | 725 (12%) | 517 (9%) | 647 (11%) | 604 (10%) | 611 (10%) | 673 (11%) | 117 (2%) |
| 0.6 - <0.8 | 183 (3%) | 111 (2%) | 53 (1%) | 64 (1%) | 183 (3%) | 95 (2%) | 145 (2%) | 58 (1%) | 56 (1%) |
| ≥0.8 | 4 (0.1%) | 2 (0%) | 0 (0%) | 5 (0.1%) | 75 (1%) | 0 (0%) | 2 (0%) | 0 (0%) | 43 (0.7%) |
