# Supplementary material for: Developing and validating an explainable digital mortality prediction tool for extremely preterm infants
Source: PLOS Digit Health. 2025 Dec 10;4(12):e0000955. doi: 10.1371/journal.pdig.0000955 (PMC12694798; doi:10.1371/journal.pdig.0000955)
Supplement: S4 Table — Regression coefficient denotes estimated change in the log odds of death when the associated predictor increases by one unit or in comparison with a reference category. NICU denotes Neonatal Intensive Care Unit. (DOCX) [file pdig.0000955.s006.docx]

# S4 Table

Table presenting the regression coefficients with 95% confidence intervals (CI) of the final logistic regression model to predict death before neonatal discharge. Regression coefficient denotes estimated change in the log odds of death when the associated predictor increases by one unit or in comparison with a reference category. NICU denotes Neonatal Intensive Care Unit.

* Reference category: ‘Male’ for sex; ‘No course’ for antenatal corticosteroids; and ‘No’ for multiple pregnancy, prolonged rupture of membranes, and born in a centre with NICU, respectively.

^1^ Restricted cubic spline terms: The four knots for the gestational age at birth were 23^+5^, 25^+3^, 26^+5^ and 27^+5^ weeks.

| **Predictor** | **Regression Coefficient (95% CI)** |
| --- | --- |
| Gestational Age at Birth^1^  Gestation1  Gestation2  Gestation3 | -0.85 (-0.95 to -0.75)  0.48 (0.22 to 0.75)  -1.59 (-2.79 to -0.40) |
| Birth Weight Z-score | -0.35 (-0.41 to -0.30) |
| Sex (Female)* | -0.23 (-0.31 to -0.15) |
| Antenatal Corticosteroids*  Incomplete course  Complete course | -0.67 (-0.81 to -0.54)  -0.87 (-0.99 to -0.75) |
| Multiple Pregnancy* | 0.18 (0.09 to 0.27) |
| Prolonged rupture of membranes* | 0.19 (0.08 to 0.30) |
| Born in a centre with NICU* | -0.17 (-0.26 to -0.09) |
| Intercept | 20.91 (18.47 to 23.36) |
