# Supplementary material for: Developing and validating an explainable digital mortality prediction tool for extremely preterm infants
Source: PLOS Digit Health. 2025 Dec 10;4(12):e0000955. doi: 10.1371/journal.pdig.0000955 (PMC12694798; doi:10.1371/journal.pdig.0000955)
Supplement: S5 Table — ROC = Receiver Operating Characteristic. 1,500 (26%) and 55 (1%) of infants with missing data on maternal ethnicity and neonatal network were excluded. (DOCX) [file pdig.0000955.s007.docx]

# S5 Table

Table describing the incidence of death and the model performance of the logistic regression approach in the ‘test’ cohort (N = 5,879) stratified by (A) maternal ethnicity (N = 4,379) and (B) neonatal network (N = 5,824). ROC = Receiver Operating Characteristic. 1,500 (26%) and 55 (1%) of infants with missing data on maternal ethnicity and neonatal network were excluded.

## (A) Maternal ethnicity

| **Characteristics** | **White (N = 2,948)** | **South Asian (N = 671)** | **Black (N = 536)** | **Other/Mixed (N = 224)** |
| --- | --- | --- | --- | --- |
| **Incidence of death, n (%)** | 551 (19) | 112 (17) | 99 (18) | 38 (17) |
| **Model performance (95% CI)** |  |  |  |  |
| **Discrimination**  Area under the ROC curve | 0.754  (0.730 to 0.777) | 0.742  (0.692 to 0.791) | 0.713  (0.655 to 0.770) | 0.759  (0.664 to 0.854) |
| **Calibration**  Calibration-in-the-large  Calibration slope | -0.02 (-0.18 to 0.15)  1.05 (0.94 to 1.17) | -0.31 (-0.65 to 0.03)  1.04 (0.79 to 1.31) | -0.53 (-0.86 to -0.20)  0.84 (0.59 to 1.09) | -0.30 (-0.85 to 0.25)  1.02 (0.65 to 1.43) |

## (B) Neonatal network

| **Model performance** | **Network 1 (N = 352)** | **Network 2 (N = 449)** | **Network 3 (N = 478)** | **Network 4 (N = 328)** | **Network 5 (N = 772)** |
| --- | --- | --- | --- | --- | --- |
| **Incidence of death, %** | 74 (21) | 67 (15) | 54 (11) | 56 (17) | 172 (22) |
| **Model performance (95% CI)** |  |  |  |  |  |
| **Discrimination**  Area under the ROC curve | 0.772  (0.708 to 0.837) | 0.759  (0.691 to 0.826) | 0.757  (0.678 to 0.836) | 0.739  (0.671 to 0.808) | 0.745  (0.702 to 0.789) |
| **Calibration**  Calibration-in-the-large  Calibration slope | 0.35 (-0.13 to 0.86)  1.26 (0.93 to 1.62) | -0.18 (-0.64 to 0.28)  1.16 (0.84 to 1.49) | -0.90 (-1.29 to -0.52)  1.08 (0.77 to 1.42) | -0.27 (-0.77 to 0.23)  1.03 (0.67 to 1.40) | 0.21 (-0.10 to 0.53)  1.07 (0.86 to 1.29) |

| **Model performance** | **Network 6 (N = 305)** | **Network 7 (N = 487)** | **Network 8 (N = 419)** | **Network 9 (N = 313)** | **Network 10 (N = 466)** |
| --- | --- | --- | --- | --- | --- |
| **Incidence of death, %** | 62 (20) | 89 (18) | 81 (19) | 50 (16) | 64 (14) |
| **Model performance (95% CI)** |  |  |  |  |  |
| **Discrimination**  Area under the ROC curve | 0.767  (0.701 to 0.834) | 0.783  (0.733 to 0.834) | 0.738  (0.675 to 0.802) | 0.758  (0.682 to 0.834) | 0.725  (0.649 to 0.801) |
| **Calibration**  Calibration-in-the-large  Calibration slope | 0.05 (-0.43 to 0.53)  1.07 (0.75 to 1.42) | -0.01 (-0.40 to 0.38)  1.19 (0.90 to 1.50) | -0.19 (-0.61 to 0.22)  0.97 (0.68 to 1.28) | -0.30 (-0.81 to 0.22)  1.03 (0.68 to 1.41) | -0.59 (-1.02 to -0.17)  0.93 (0.64 to 1.23) |

| **Model performance** | **Network 11 (N = 262)** | **Network 12 (N = 665)** | **Network 13 (N = 528)** |
| --- | --- | --- | --- |
| **Incidence of death, %** | 66 (25) | 157 (24) | 108 (20) |
| **Model performance (95% CI)** |  |  |  |
| **Discrimination**  Area under the ROC curve | 0.734  (0.657 to 0.811) | 0.755  (0.711 to 0.798) | 0.713  (0.660 to 0.766) |
| **Calibration**  Calibration-in-the-large  Calibration slope | 0.25 (-0.25 to 0.76)  0.98 (0.66 to 1.32) | 0.26 (-0.07 to 0.59)  1.00 (0.79 to 1.22) | -0.16 (-0.55 to 0.23)  0.84 (0.59 to 1.09) |
