# Supplementary material for: Developing and validating an explainable digital mortality prediction tool for extremely preterm infants
Source: PLOS Digit Health. 2025 Dec 10;4(12):e0000955. doi: 10.1371/journal.pdig.0000955 (PMC12694798; doi:10.1371/journal.pdig.0000955)

# S3 Fig

Decision curve analysis of the logistic regression approach in the ‘test’ cohort (N = 5,879) stratified by (A) maternal ethnicity (N = 4,379) and (B) neonatal network (N = 5,824).

## (A) Maternal ethnicity


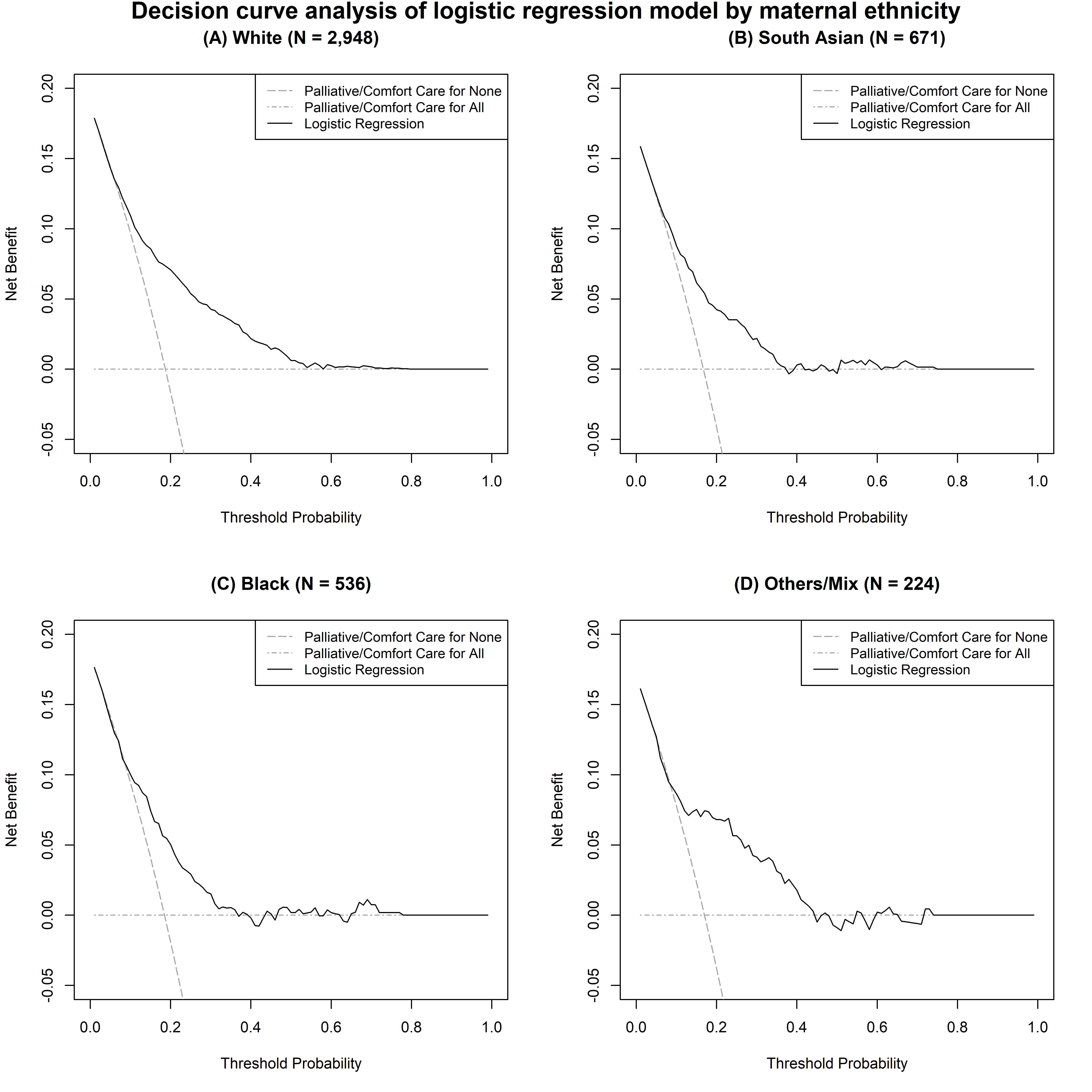


## (B) Neonatal network


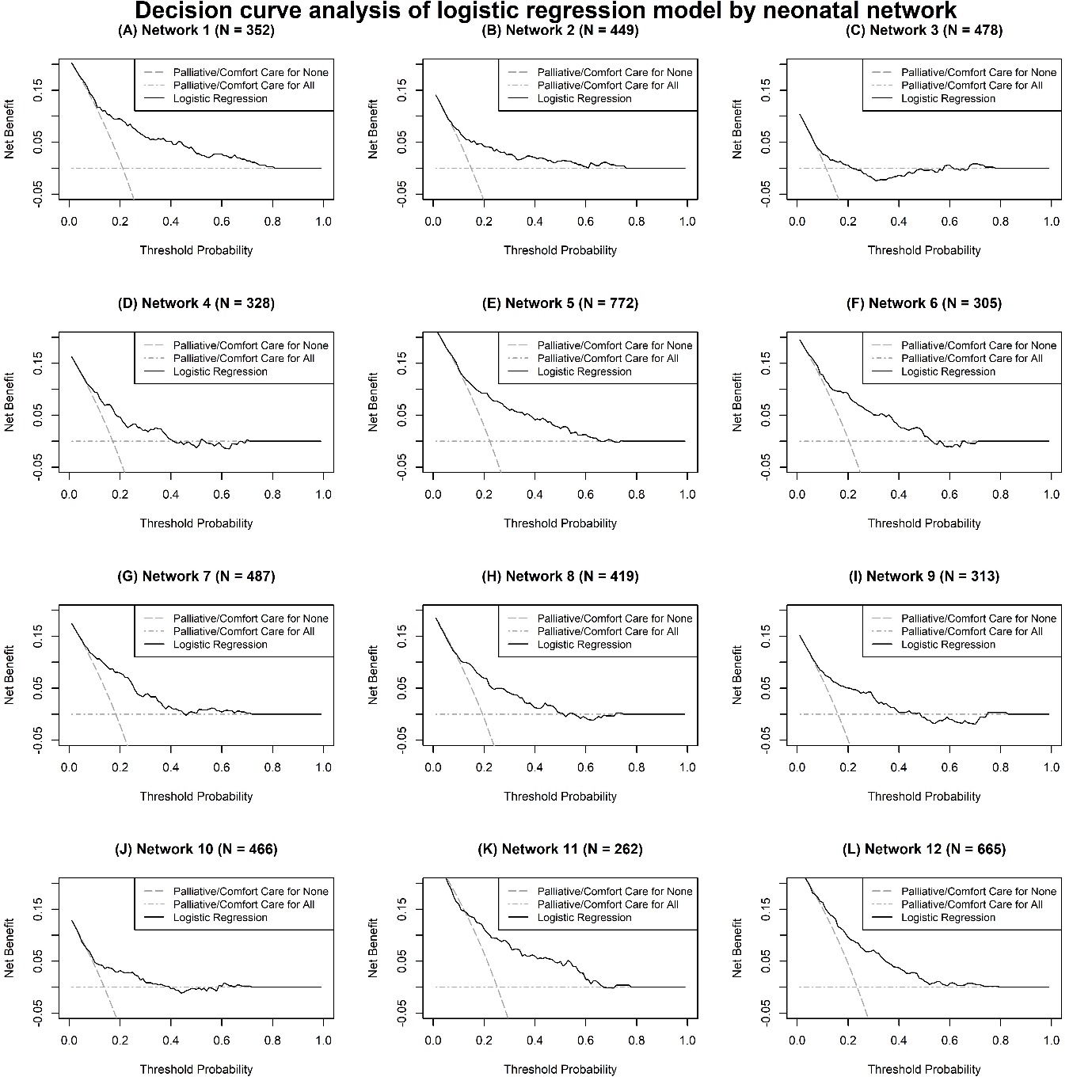


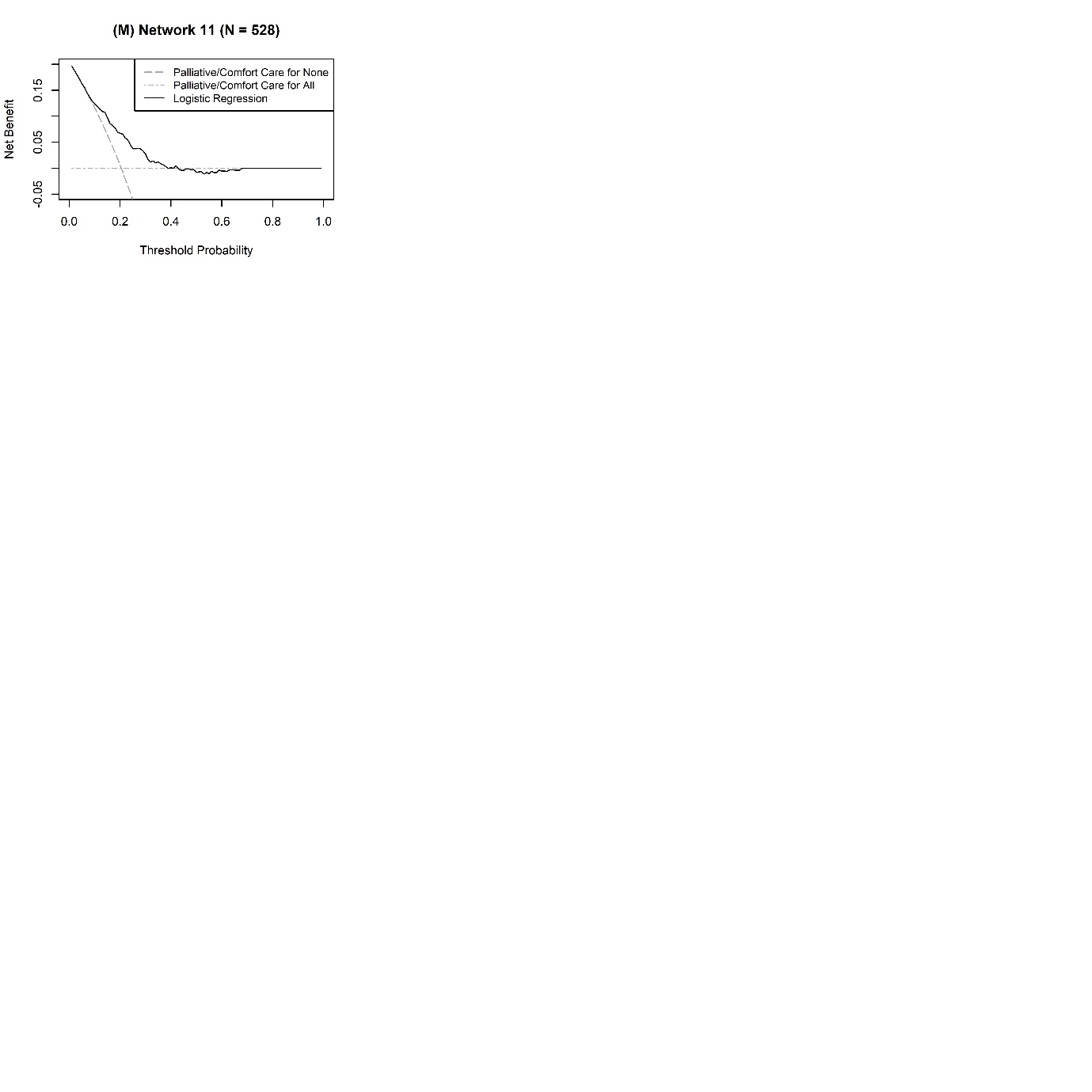

Supplement: S3 Fig — (DOCX) [file pdig.0000955.s009.docx]
