# Supplementary material for: Developing and validating an explainable digital mortality prediction tool for extremely preterm infants
Source: PLOS Digit Health. 2025 Dec 10;4(12):e0000955. doi: 10.1371/journal.pdig.0000955 (PMC12694798; doi:10.1371/journal.pdig.0000955)
Supplement: S6 Table — (DOCX) [file pdig.0000955.s010.docx]

# S6 Table

Table describing the definition of variables extracted from the National Neonatal Research Database.

| **Variable** | **Data Items** |  |
| --- | --- | --- |
| **Predictors** |  |  |
| Gestational age | - Data extracted from “GESTATIONDAYS” and “GESTATIONWEEKS” variables in the “EPISODES” dataset. - Continuous in days |  |
| Birth weight z score | **Definition**: Birth weight z score derived from the UK-WHO growth chart(62).   - Data extracted from “BIRTHWEIGHT”, “GESTATIONDAYS”, “GESTATIONWEEKS” and “SEX” variables in the “EPISODES” dataset. - Continuous |  |
| Sex | - Data extracted from “SEX” variable in the “EPISODES” dataset. - Dichotomous (Male– 0/Female – 1) |  |
| Born in a maternity centre with a co-located neonatal intensive care unit (NICU) | **Definition**: Infant delivered in a maternity unit with a co-located NICU.   - Data extracted from “pobndaucode” variable in the “EPISODES” dataset and the “UnitLevel” variable in the “UNITLEVELSANDNDAUCODES” dataset - Dichotomous (Yes – 1/No – 0) |  |
| Multiple pregnancy | - Data extracted from “fetusnumber” variable of more than 1 in the “EPISODES” dataset. - Dichotomous (Yes – 1/No – 0) |  |
| Antenatal corticosteroids | **Definition**: Any exposure to antenatal corticosteroids received before delivery   - Data extracted from “steroidsantenatalcourses” variable in the “EPISODES” dataset. - Categorical (Complete – 2/Incomplete – 1/No course – 0) |  |
| Prolonged rupture of membranes | **Definition**: Rupture of membranes for more than 18 hours.   - Data extracted from “ROMTIMEANON” variable or “Prolonged rupture membranes” response from the “problemsduringpregnancy” variable in the “EPISODES” dataset. - Dichotomous (Yes – 1/No – 0) |  |
| Maternal chorioamnionitis | **Definition**: Clinically suspected maternal chorioamnionitis by the obstetrics team.   - Data extracted from “Chorioamnionitis” response from the “problemsduringpregnancy” variable in the “EPISODES” dataset as well as the “diagnosisatadmission” and “principaldiagnosisatdischarge” variables in the “EPISODES” dataset for “Chorioamnionitis”. - Dichotomous (Yes – 1/No – 0) |  |
| Congenital anomalies | **Definition**: Major congenital anomalies as defined in the European registry of congenital anomalies(37) and the previous publication by the research group(63).   - Data extracted from “diagnosisatadmission” and “principaldiagnosisatdischarge” variables in the “EPISODES” dataset. - Dichotomous (Yes – 1/No – 0) |  |
| **Outcome** | |  |
| Death | **Definition**: Death before discharge from the neonatal unit.   - Data extracted from “dateofdeath” and “dischargedestination” variables from the “EPISODES” dataset. - Dichotomous (Yes – 1/No – 0) |  |
| **Other variables** | | |
| Birth year | - Data extracted from BIRTHYEAR variable in the “EPISODES” dataset. - Continuous |  |
| Maternal ethnicity | - Data extracted from “MUMETHNICITY” variable in the “EPISODE” dataset. - Categorical (White, South Asian, Black or Others/Mix) |  |
| Neonatal network at birth | - Data extracted from “pobndaucode” variable in the “EPISODES” dataset and the “NEONATAL_ODN” variable in the “ODNMAPPINGANDNDAUCODES” dataset. - Categorical (13 networks) |  |

## References

1. Royal College of Paediatrics and Child Health. UK-WHO growth charts - neonatal and infant close monitoring (NICM). <https://www.rcpch.ac.uk/resources/uk-who-growth-charts-neonatal-infant-close-monitoring-nicm>. Date last accessed: March 01 2021.

2. European Surveillance of Congenital Anomalies (EUROCAT). EUROCAT Guide 1.4 and Reference Documents. 2013. <http://www.eurocat-network.eu/>. [Date last accessed: February 12 2020].

3. Sand L, Szatkowski L, Kwok TC, et al. Observational cohort study of changing trends in non-invasive ventilation in very preterm infants and associations with clinical outcomes. *Arch Dis Child Fetal Neonatal Ed* 2021.
