# Supplementary material for: Developing and validating an explainable digital mortality prediction tool for extremely preterm infants
Source: PLOS Digit Health. 2025 Dec 10;4(12):e0000955. doi: 10.1371/journal.pdig.0000955 (PMC12694798; doi:10.1371/journal.pdig.0000955)
Supplement: S7 Table — The list was accessed from https://www.imperial.ac.uk/neonatal-data-analysis-unit/neonatal-data-analysis-unit/list-of-national-neonatal-units/ on 06/01/2022. (DOCX) [file pdig.0000955.s011.docx]

# S7 Table

Participating neonatal units in England and Wales and their respective lead clinicians. The list was accessed from <https://www.imperial.ac.uk/neonatal-data-analysis-unit/neonatal-data-analysis-unit/list-of-national-neonatal-units/> on 06/01/2022.

| **Institution** | **Lead clinician** |
| --- | --- |
| Airedale General Hospital | Dr Matthew Babirecki |
| Arrowe Park Hospital | Dr Anand Kamalanathan |
| Barnet Hospital | Dr Tim Wickham |
| Barnsley District General Hospital | Dr Kavi Aucharaz |
| Basildon Hospital | Dr Aashish Gupta |
| Basingstoke & North Hampshire Hospital | Dr Nicola Paul |
| Bassetlaw District General Hospital | Dr L M Wong |
| Bedford Hospital | Dr Anita Mittal |
| Birmingham City Hospital | Dr Lindsay Halpern |
| Birmingham Heartlands Hospital | Dr Pinki Surana |
| Birmingham Women's Hospital | Dr Matt Nash |
| Bradford Royal Infirmary | Dr Sam Wallis |
| Broomfield Hospital, Chelmsford | Dr Ahmed Hassan |
| Calderdale Royal Hospital | Dr Karin Schwarz |
| Chelsea & Westminster Hospital | Dr Shu-Ling Chuang |
| Chesterfield & North Derbyshire Royal Hospital | Dr Aiwyne Foo |
| Colchester General Hospital | Dr Jo Anderson |
| Conquest Hospital | Dr Graham Whincup |
| Countess of Chester Hospital | Dr Stephen Brearey |
| Croydon University Hospital | Dr Morris |
| Croydon University Hospital | Dr Srirambhatla |
| Cumberland Infirmary | Dr Yee Aung |
| Darent Valley Hospital | Dr Abdul Hasib |
| Darlington Memorial Hospital | Dr Mehdi Garbash |
| Derriford Hospital | Dr Alex Allwood |
| Diana Princess of Wales Hospital | Dr Pauline Adiotomre |
| Doncaster Royal Infirmary | Dr Nigel Brooke |
| Dorset County Hospital | Dr Abby Deketelaere |
| East Surrey Hospital | Dr Abdul Khader |
| Epsom General Hospital | Dr Sonia Spathis |
| Frimley Park Hospital | Dr Sanghavi Rekha |
| Furness General Hospital | Dr Anas Olabi |
| George Eliot Hospital | Dr Mukta Jain |
| Glan Clwyd Hospital | Dr Ian Barnard |
| Glangwili General Hospital | Dr Prem Pitchaikani |
| Gloucester Royal Hospital | Dr Jennifer Holman |
| Good Hope Hospital | Dr Pinki Surana |
| Great Western Hospital | Dr Stanley Zengeya |
| Guy's & St Thomas' Hospital | Dr Geraint Lee |
| Harrogate District Hospital | Dr Sobia Balal |
| Hereford County Hospital | Dr Cath Seagrave |
| Hillingdon Hospital | Dr Tristan Bate |
| Hinchingbrooke Hospital | Dr Hilary Dixon |
| Homerton Hospital | Dr Narendra Aladangady |
| Hull Royal Infirmary | Dr Hassan Gaili |
| Ipswich Hospital | Dr Matthew James |
| James Cook University Hospital | Dr M Lal |
| James Paget Hospital | Dr Ambadkar |
| Kettering General Hospital | Dr Poornima Pandey |
| Kings College Hospital | Dr Ravindra Bhat |
| King's Mill Hospital | Dr Simon Rhodes |
| Kingston Hospital | Dr Jonathan Filkin |
| Lancashire Women and Newborn Centre | Dr Savi Sivashankar |
| Leeds Neonatal Service | Dr Lawrence Miall |
| Leicester General Hospital | Dr Jonathan Cusack |
| Leicester Royal Infirmary | Dr Venkatesh Kairamkonda |
| Leighton Hospital | Dr Michael Grosdenier |
| Lincoln County Hospital | Dr Ajay Reddy |
| Lister Hospital | Dr J Kefas |
| Liverpool Women's Hospital | Dr Christopher Dewhurst |
| Luton & Dunstable Hospital | Dr Jennifer Birch |
| Macclesfield District General Hospital | Dr Gail Whitehead |
| Manor Hospital | Dr Ashok Karupaiah |
| Medway Maritime Hospital | Dr Ghada Ramadan |
| Milton Keynes General Hospital | Dr I Misra |
| Musgrove Park Hospital | Dr Chris Knight |
| New Cross Hospital | Dr Matt Nash |
| Newham General Hospital | Dr Imdad Ali |
| Nobles Hospital | Dr Prakash Thiagarajan |
| Norfolk & Norwich University Hospital | Dr Muthukumar |
| North Devon District Hospital | Dr Michael Selter |
| North Manchester General Hospital | Dr Ajit Mahaveer |
| North Middlesex University Hospital | Dr Neeraj Jain |
| Northampton General Hospital | Dr Subodh Gupta |
| Northumbria Specialist Emergency Care Hospital | Jess Reynolds |
| Northwick Park Hospital | Dr Richard Nicholl |
| Nottingham City Hospital | Dr Steven Wardle |
| Nottingham University Hospital (QMC) | Dr Steven Wardle |
| Ormskirk District General Hospital | Dr Andreea Bontea |
| Oxford University Hospitals, John Radcliffe Hospital | Dr Eleri Adams |
| Peterborough City Hospital | Dr Katharine McDevitt |
| Pilgrim Hospital | Dr Ajay Reddy |
| Pinderfields General Hospital (Pontefract General Infirmary) | Dr David Gibson |
| Poole General Hospital | Prof Minesh Khashu |
| Prince Charles Hospital | Dr Iyad Al-Muzaffar |
| Princess Alexandra Hospital | Dr Chinnappa Reddy |
| Princess Anne Hospital | Dr Mark Johnson |
| Princess of Wales Hospital | Dr Kate Creese |
| Princess Royal Hospital | Dr P Amess |
| Princess Royal Hospital (previously Royal Shrewsbury Hospital) | Dr Deshpande |
| Princess Royal University Hospital | Dr Elizabeth Sleight |
| Queen Alexandra Hospital | Dr Charlotte Groves |
| Queen Charlotte's Hospital | Dr Lidia Tyszcuzk |
| Queen Elizabeth Hospital, Gateshead | Dr Anne Dale |
| Queen Elizabeth Hospital, King's Lynn | Dr Glynis Rewitzky |
| Queen Elizabeth Hospital, Woolwich - see notes | Dr Olutoyin Banjoko |
| Queen Elizabeth the Queen Mother Hospital | Dr Bushra Abdul-Malik |
| Queen's Hospital, Burton on Trent | Dr Dominic Muogbo |
| Queen's Hospital, Romford | Dr Khalid Mannan |
| Queen's Hospital, Romford 2 | Dr Khalid Mannan |
| Rosie Maternity Hospital, Addenbrookes | Dr Angela D'Amore |
| Rotherham District General Hospital | Dr Soma Sengupta |
| Royal Albert Edward Infirmary | Dr Christos Zipitis |
| Royal Berkshire Hospital | Dr Peter De Halpert |
| Royal Bolton Hospital | Dr Paul Settle |
| Royal Cornwall Hospital | Dr Paul Munyard |
| Royal Derby Hospital | Dr John McIntyre |
| Royal Devon & Exeter Hospital | Dr Chrissie Oliver |
| Royal Gwent Hospital | Dr Sunil Reddy |
| Royal Hampshire County Hospital | Dr Lucinda Winckworth |
| Royal Lancaster Infirmary | Dr Joanne Fedee |
| Royal Oldham Hospital | Dr Natasha Maddock |
| Royal Preston Hospital | Dr Richa Gupta |
| Royal Stoke University Hospital | Dr Jyoti Kapur |
| Royal Surrey County Hospital | Dr Ben Obi |
| Royal Sussex County Hospital | Dr P Amess |
| Royal United Hospital | Dr Stephen Jones |
| Royal Victoria Infirmary | Dr Naveen Athiraman |
| Russells Hall Hospital | Dr Chandan Gupta |
| Salisbury District Hospital | Dr Jim Baird |
| Scarborough General Hospital | Dr Kirsten Mack |
| Scunthorpe General Hospital | Dr Pauline Adiotomre |
| Singleton Hospital | Dr Arun Ramachandran |
| Southend Hospital | Dr Vineet Gupta |
| Southmead Hospital | Dr Faith Emery |
| St George's Hospital | Dr Charlotte Huddy |
| St Helier Hospital | Dr Ralf Hartung |
| St Mary's Hospital, IOW | Dr Akinsola Ogundiya |
| St Mary's Hospital, London | Dr Lidia Tyszcuzk |
| St Mary's Hospital, Manchester | Dr Ngozi Edi-Osagie |
| St Michael's Hospital | Dr Pamela Cairns |
| St Peter's Hospital | Dr Peter Martin |
| St Richard's Hospital | Dr Victoria Sharp |
| Stepping Hill Hospital | Dr Carrie Heal |
| Stoke Mandeville Hospital | Dr Sanjay Salgia |
| Sunderland Royal Hospital | Dr Majd Abu-Harb |
| Tameside General Hospital | Dr Jacqeline Birch |
| The Grange University Hospital | Dr Sunil Reddy |
| The Jessop Wing, Sheffield | Dr Porus Bastani |
| The Royal Free Hospital | Dr Marice Theron |
| The Royal London Hospital - Constance Green | Dr Vadivelam Murthy |
| Torbay Hospital | Dr Siba Paul |
| Tunbridge Wells Hospital | Dr Hamudi Kisat |
| University College Hospital | Dr Giles Kendall |
| University Hospital Coventry | Dr Puneet Nath |
| University Hospital Lewisham | Dr Ozioma Obi |
| University Hospital of North Durham | Dr Mehdi Garbash |
| University Hospital of North Tees | Dr Hari Kumar |
| University Hospital of Wales | Dr Nitin Goel |
| Victoria Hospital, Blackpool | Dr Chris Rawlingson |
| Warrington Hospital | Dr Delyth Webb |
| Warwick Hospital | Dr Bird |
| Watford General Hospital | Dr Sankara Narayanan |
| West Cumberland Hospital | Dr Yee Aung |
| West Middlesex University Hospital | Dr Eleanor Hulse |
| West Suffolk Hospital | Dr Ian Evans |
| Wexham Park Hospital | Dr Sanjay Jaisal |
| Whipps Cross University Hospital | Dr Caroline Sullivan |
| Whiston Hospital | Dr Ros Garr |
| Whittington Hospital | Dr Wynne Leith |
| William Harvey Hospital | Dr Vimal Vasu |
| Withybush Hospital | Dr Vishwa Narayan |
| Worcestershire Royal Hospital | Dr Liza Harry |
| Worthing Hospital | Dr Katia Vamvakiti |
| Wrexham Maelor Hospital | Dr Brendan Harrington |
| Wythenshawe Hospital | Dr Ngozi Edi-Osagie |
| Yeovil District Hospital | Dr Megan Eaton |
| York District Hospital | Dr Sundeep Sandhu |
| Ysbyty Gwynedd | Dr Mike Cronin |
