# Supplementary material for: Developing and validating an explainable digital mortality prediction tool for extremely preterm infants
Source: PLOS Digit Health. 2025 Dec 10;4(12):e0000955. doi: 10.1371/journal.pdig.0000955 (PMC12694798; doi:10.1371/journal.pdig.0000955)
Supplement: Attachment — Submitted filename: plos digital health review.pdf [file pdig.0000955.s012.pdf]

This manuscript describes the development and validation of an explainable mortality prediction tool for preterm infants using a large dataset, and an external validation cohort. The authors used and compared nine machine learning approaches and chose a logistic regression model for its interpretability and comparable performance. An online tool is made available for clinical use.

Major comments:

1. The authors are recommended to include a section to clearly introduce the data preparation, training and testing process, inputs and outputs, etc. Clarify how did they prevent data leakage, what kind of validation methods were used…better to describe with a figure.
2. Please identify the internal and external validations in this study.
3. Please emphasize the knowledge gaps and the novelties in this study.
4. The model showed weaker performance in certain subgroups (e.g., infants of Black mothers). How should clinicians account for this when using the tool? Could the authors propose mitigation strategies or monitoring plans to avoid exacerbating health inequities?
5. The PLUSS cohort represents trial-enrolled infants who may not be fully representative of real-world neonatal populations. Could the authors clarify how this might limit generalizability? Are there plans for broader external validations?
6. The manuscript notes overestimation of mortality in certain networks and subgroups. Could the authors provide more details on recalibration strategies or whether periodic recalibration is planned?
7. Mortality prediction in perinatal counseling is sensitive. How do the authors propose this tool be used in shared decision-making without creating undue bias toward particular care pathways?
8. Mortality before discharge is a relevant but limited endpoint. Parents often wish to know about long-term neurodevelopmental outcomes. Are there plans to extend the model to predict major morbidities or neurodevelopmental outcomes?

Minor comments:

1. Please introduce why logistic regression was chosen over slightly better-performing models (e.g., extreme gradient boosting).
2. Clarify handling of missing data in predictor variables.
3. Why did the authors only use AUC for model metrics? How about other metrics? Please explain.
