# Supplementary material for: Developing and validating an explainable digital mortality prediction tool for extremely preterm infants
Source: PLOS Digit Health. 2025 Dec 10;4(12):e0000955. doi: 10.1371/journal.pdig.0000955 (PMC12694798; doi:10.1371/journal.pdig.0000955)
Supplement: Attachment — Submitted filename: Response to reviewers.docx [file pdig.0000955.s014.docx]

*Thank you for the opportunity to revise our manuscript based on the crucial feedback from the three peer reviewers. Below are our point-by-point responses to the feedback in blue italic text.*

**Reviewers' Comments:**

**Reviewer #1**

The current manuscript does not clearly articulate the significance, objectives, and clinical implications of mortality prediction. While the authors mention risk factors and existing prediction models, there is no systematic discussion of why individualized mortality prediction is needed or how such predictions can provide practical value for family counselling and clinical practice.

*Thank you for the suggestion. We have added an ”Individualised prediction” sub-section within the discussion to explain this further, highlighting the need for individualised prediction and how it could support perinatal counselling, shared clinical decision-making, as well as future research. The sub-section also discussed the novelty of the study and how the tool could be used in clinical practice, alongside its limitations.*

Moreover, the main technical challenges related to methodology, data, and clinical application are not clearly summarized. I recommend adding relevant content to help readers better understand the innovation and real-world significance of this research.

*Thank you for the suggestion. We have highlighted and explained the main technical challenges further in the “Challenges/limitations” sub-section within the discussion. Relevant headings are used to improve readability of the paper.*

The authors state in the Introduction that a major limitation of current mortality prediction models for extremely preterm infants is their lack of actual clinical implementation, which is a key challenge in the field. However, although this study has developed and validated a new prediction tool, it does not evaluate its deployment, user feedback, or support for real-world clinical decision-making. Thus, while there are advances in modeling and data scale, the work does not address the critical bottleneck of clinical translation. I suggest the authors clarify this limitation in the Discussion and provide more concrete recommendations for future efforts to facilitate clinical implementation.

*Thank you for the suggestion. We have carried out two focus groups with parents of extreme preterm infants to co-design the online tool as the first step in facilitating clinical implementation. This is explained in the methods section. We will continue to evaluate the usability of the tool once it is deployed clinically and assess its impact on real-world clinical decision-making. This has been expanded further in the discussion section.*

The use of SHAP and LIME to illustrate feature contributions is primarily limited to presenting the statistical importance of input variables. It should be noted that such 'explainability' is confined to variable ranking at the technical level and does not address the causal reasoning, decision logic, or medical context that clinicians require in practice. Therefore, the model’s explainability remains largely technical and falls short of true clinical interpretability and trustworthiness. I recommend that the authors expand their discussion of the current state and limitations of model explainability, especially in relation to the real needs of clinicians and families and explore how to enhance clinical utility and interpretability in future work.

*Thank you for the important point. We have included this in the discussion section.*

The potential negative impacts of model deployment, such as families becoming overly reliant on model predictions, resulting in pessimistic or inappropriate decision-making, or the risk of doctor-patient disputes and inequitable resource allocation, are not sufficiently addressed. The manuscript only briefly mentions the need for further evaluation, lacking substantive discussion or risk warnings.
*Thank you for the important point. We have included this in the ”Individualised prediction” sub-section within the discussion. We have highlighted that the predictions made by the tool would need to be interpreted based on the clinical condition of the infant, with clinical input from healthcare professionals. It should be viewed as part of an armoury of investigation results that healthcare professionals have to hand to support shared decision-making, rather than making a clinical decision solely based on the tool.*

The modeling process is described in very general terms; while nine machine learning methods are compared, the specific implementation details, parameter settings, and feature engineering procedures are not disclosed. There is no clear description of model training, validation, or tuning processes, including how the training/validation/test sets were divided, the cross-validation strategies used, whether tuning was automated, or whether measures such as early stopping and regularization were employed.
*The training/validation/test dataset division was explained under the “Model development and internal validation” subsection of the method. To avoid detraction from the main study findings, a detailed description of the modelling process for each of the nine machine learning methods is given in the supplementary information as S1 file.*

While the authors mention the use of complete case analysis for missing data, there is no information on the missingness rates for each variable, or on whether sensitivity analyses or imputation were performed.
*The missing rate for each variable is provided in Table 1, with the characteristics of infants with missing data described in Supplementary Table 1. Imputation/sensitivity analysis was not used as missing data only accounted for a small (6%) proportion of the overall cohort, and there was no major difference in basic demographics of the infants between those with and without missing data (Supplementary Table 1).*

it's recommend providing analysis code or a detailed modeling protocol as supplementary material to enhance the study’s reproducibility.
*To avoid detraction from the main study findings, a detailed description of the modelling process is given in the supplementary information as S1 file.*

The results show that model calibration or performance declines in certain subgroups (e.g., infants of Black mothers, certain regions). The authors should further analyze the reasons and potential impact, and discuss possible improvement strategies, with appropriate additions to the Discussion.
*We have highlighted the challenges in interpreting the performance in our subgroup analysis in the “Challenges/limitations” section of the discussion, with potential reasons for its variable performance and future work to monitor the tool performance across different subgroups. Further analysis of this is unfortunately not possible and beyond the scope of the current work.*

It should be explicitly stated that this model does not include infants who died before NICU admission, which may result in underestimating mortality rates among the highest-risk population.

*This has been further highlighted in the discussion section. Thank you.*

I also recommend discussing the limitations of using only perinatal variables and the potential for incorporating dynamic clinical data, postnatal complications, and multi-omics biomarkers in future models.

*This has been further highlighted in the discussion section as suggested. Thank you.*

**Reviewer #2**

This study addresses the important and challenging task of mortality prediction in extremely preterm infants using routinely collected electronic health record data. The authors compared nine machine learning approaches and developed an explainable tool based on stepwise backward logistic regression. The topic is clinically relevant and the study design is rigorous; however, some methodological clarifications and additional analyses are needed to strengthen the manuscript.
*Thank you.*

Major Weakness:
1. Please provide a clear description of how each input feature was numerically encoded. For example, specify that Sex was coded as 1 (Male) and 0 (Female). Such details will help readers interpret the SHAP plots more accurately.

*This has been highlighted as the legend in Fig 1 as well as in Supplementary Table 5.*

2. The font size of the feature names and the numeric values on the x-axis in the SHAP plots should be increased or otherwise adjusted to improve clarity and readability.

*Thank you. This has been increased as suggested.*

3. In Table 2, the values for Calibration-in-the-large and Calibration slope are presented. Please explain how these metrics were derived in the methods section so that readers can better understand their calculation and interpretation.

*Thank you for the suggestion. We have provided the reference(1) of how the calibration-in-the-large and calibration slope were derived and their interpretation in the methods section.*

4. According to the calibration plot, overfitting does not appear to be a major issue across the machine learning methods tested. However, for traditional machine learning methods, calibration analysis may be of limited relevance since these approaches are inherently less prone to overfitting. Given that the AUROC values are only around 0.75, I encourage the authors to provide the distribution of predicted risks in the test set (e.g., the number of cases with predicted probability >0.8, 0.6–0.8, 0.4–0.6, etc.). This will help readers assess the practical utility of the models in real-world decision-making. If the number of high-confidence predictions (e.g., >0.8) is very small and most predictions cluster around 0.5, the limitations of the approach should be discussed more explicitly.

*Thank you for raising this important point. As suggested, most of the predicted risks clustered around <0.2. This may be due to the imbalanced dataset or models being too conservative due to a lack of postnatal clinical data beyond the perinatal period. We have included this in the discussion section.*

**Reviewer #3**

This manuscript describes the development and validation of an explainable mortality prediction tool for preterm infants using a large dataset, and an external validation cohort. The authors used and compared nine machine learning approaches and chose a logistic regression model for its interpretability and comparable performance. An online tool is made available for clinical use.

Major comments:

1. The authors are recommended to include a section to clearly introduce the data preparation, training and testing process, inputs and outputs, etc. Clarify how did they prevent data leakage, what kind of validation methods were used…better to describe with a figure.

*To avoid detraction from the main study findings, a detailed description of the data preparation and modelling process is given in the supplementary information as S1 file, alongside the data items used to extract the variables included in Supplementary Table 5. A figure was also added to S1 file as suggested.*

2. Please identify the internal and external validations in this study.

*Thank you. We have a subsection for internal and external validation in the results and method sections. Temporal internal validation was used. Data from 2018 to 2020 was set aside to internally validate the study. The study was externally validated in a different population from a clinical trial (PLUSS trial).*

3. Please emphasize the knowledge gaps and the novelties in this study.

*Thank you. We have highlighted this in the “Individualised prediction” section in Discussion. The novelty of the study is an online tool providing personalised mortality prediction using population-based real-world clinical data, with variable importance provided for each individual prediction. The tool was also co-designed with parents of extremely preterm infants as a first step towards clinical implementation.*

4. The model showed weaker performance in certain subgroups (e.g., infants of Black mothers). How should clinicians account for this when using the tool? Could the authors propose mitigation strategies or monitoring plans to avoid exacerbating health inequities?

*We have highlighted the challenges in interpreting the performance in our subgroup analysis in the “Challenges/limitations” section of the discussion, with potential reasons for its variable performance and future work to monitor the tool performance across different subgroups. Caution is needed when using the tool in the subgroups with poor performance. Predictions provided by the tool should be considered within the clinical context.*

5. The PLUSS cohort represents trial-enrolled infants who may not be fully representative of real-world neonatal populations. Could the authors clarify how this might limit generalizability? Are there plans for broader external validations?

*The tool was developed in a population-based dataset in England and Wales, ensuring it is generalisable to the UK population, which was the initial target population of the tool. As mentioned, the PLUSS cohort may not be fully representative due to the strict eligibility criteria for clinical trial recruitment. The PLUSS cohort likely represents a slightly more restricted population with more immature and sicker infants. However, infants were recruited from four different countries, exploring the applicability of the model outside England and Wales. This has been explained in the discussion section. Once the tool is published and available online, we encourage collaboration with other researchers to externally validate the tool further.*

6. The manuscript notes overestimation of mortality in certain networks and subgroups. Could the authors provide more details on recalibration strategies or whether periodic recalibration is planned?

*We have highlighted the challenges in interpreting the performance in our subgroup analysis in the “Challenges/limitations” section of the discussion, with potential reasons for its overestimation of mortality and future work to periodically monitor the performance across different subgroups. Caution is needed when using the tool in these subgroups, and the output of the tool should be considered within the clinical context of the infant.*

7. Mortality prediction in perinatal counseling is sensitive. How do the authors propose this tool be used in shared decision-making without creating undue bias toward particular care pathways?

*Thank you for raising this important point. We have explained this in the “Individualised prediction” in the discussion.*

8. Mortality before discharge is a relevant but limited endpoint. Parents often wish to know about long-term neurodevelopmental outcomes. Are there plans to extend the model to predict major morbidities or neurodevelopmental outcomes?

*Thank you for raising this important point. We have added our plan to extend the tool to predict other morbidities in the discussion section.*

Minor comments:

1. Please introduce why logistic regression was chosen over slightly betterperforming models (e.g., extreme gradient boosting).

*The logistic regression algorithm was chosen due to its familiarity among healthcare professionals and ease of interpretation, with a greater level of interpretability. This is explained in the results and discussion sections.*

2. Clarify handling of missing data in predictor variables.

*Complete case analysis was used.* This is described in the methods section. *The missing rate for each variable is provided in Table 1, with the characteristics of infants with missing data described in Supplementary Table 1. Imputation/sensitivity analysis was not used as missing data only accounted for a small (6%) proportion of the overall cohort, and there was no major difference in basic demographics of the infants between those with and without missing data (Supplementary Table 1).*

3. Why did the authors only use AUC for model metrics? How about other metrics? Please explain

*Model performance was assessed in three domains of (i) discrimination using the area under the receiver operating characteristic curve; (ii) calibration using the calibration plot, calibration-in-the-large, calibration slope; and (iii) utility measure using decision curve analysis. This is based on published guidance(2) and is described in the methods section.*

***References***

*1.	Huang Y, Li W, Macheret F, Gabriel RA, Ohno-Machado L. A tutorial on calibration measurements and calibration models for clinical prediction models. J Am Med Inform Assoc. 2020;27(4):621-33.*

*2.	Collins GS, Dhiman P, Ma J, Schlussel MM, Archer L, Van Calster B, et al. Evaluation of clinical prediction models (part 1): from development to external validation. BMJ. 2024;384:e074819.*
